# A Simple Treatment for Post-Partum Hæmorrhage

**Published:** 1914-06-06

**Authors:** 


### A Simple Treatment for Post-Partum Hæmorrhage.

An easy method of treating severe *post-partum* hæmorrhage which the usual methods have failed to check is recommended by Fieux in a Bordeaux medical paper, quoted in the *Medical Review*. It consists simply in placing the patient in the Trendelenburg position, whereby the hæmorrhage is instantly checked. If this observation is proved to be correct by independent investigators, the simplicity of the treatment should serve to make it popular. The theoretical explanation of the method is less simple. Fieux rejects the view that the uterus, in sinking bodily towards the diaphragm in the Trendelenburg posture, stretches or kinks the uterine vessels sufficiently to cause hæmostasis of them. Nor does he think that the relief of cerebral anæmia causes reflex contraction of the uterus. Probably he is right in rejecting both suppositions. He prefers to regard the beneficial result as due to stimulation of the interior of the uterus by the weight of the column of blood in it and in the vagina, thrown against it by the posture.

